# Regulation of gene expression by altered promoter methylation using a CRISPR/Cas9-mediated epigenetic editing system

**DOI:** 10.1038/s41598-019-48130-3

**Published:** 2019-08-19

**Authors:** Jeong Gu Kang, Jin Suk Park, Jeong-Heosn Ko, Yong-Sam Kim

**Affiliations:** 1 Genome Editing Research Center, Korea Research Institute of Bioscience and Biotechnology (KRIBB), 125 Gwahak-ro, Yuseong-gu, Daejeon 34141 Korea; 2 Department of Biomolecular Science, KRIBB School of Bioscience, Korea University of Science and Technology (UST), 217 Gajeong-ro, Yuseong-gu, Daejeon 34113 Korea

**Keywords:** DNA methylation, Molecular engineering

## Abstract

Despite the increased interest in epigenetic research, its progress has been hampered by a lack of satisfactory tools to control epigenetic factors in specific genomic regions. Until now, many attempts to manipulate DNA methylation have been made using drugs but these drugs are not target-specific and have global effects on the whole genome. However, due to new genome editing technologies, potential epigenetic factors can now possibly be regulated in a site-specific manner. Here, we demonstrate the utility of CRISPR/Cas9 to modulate methylation at specific CpG sites and to elicit gene expression. We targeted the murine Oct4 gene which is transcriptionally locked due to hypermethylation at the promoter region in NIH3T3 cells. To induce site-specific demethylation at the Oct4 promoter region and its gene expression, we used the CRISPR/Cas9 knock-in and CRISPR/dCas9-Tet1 systems. Using these two approaches, we induced site-specific demethylation at the Oct4 promoter and confirmed the up-regulation of Oct4 expression. Furthermore, we confirmed that the synergistic effect of DNA demethylation and other epigenetic regulations increased the expression of Oct4 significantly. Based on our research, we suggest that our proven epigenetic editing methods can selectively modulate epigenetic factors such as DNA methylation and have promise for various applications in epigenetics.

## Introduction

Epigenetics is the study of the heritable changes in gene expression without altering the underlying DNA sequences^1–3^. Epigenetic changes can be caused by age, disease state, and environmental conditions^4,5^. DNA methylation, histone modification and non-coding RNA associated gene silencing have been reported as major epigenetic mechanisms^6,7^. DNA methylation occurs by the addition of a methyl (CH_3_) group at the 5-carbon of the cytosine ring of DNA, thereby often regulating the expression of genes^8–10^. DNA methylation is essential for numerous cellular processes, including genomic imprinting, X-chromosome inactivation, chromosome stabilizing, and repression of transposable elements, aging, and carcinogenesis^11–13^. Epigenetic studies have uncovered valuable evidence on DNA methylation, but further studies still need to be done in this field.

According to previous reports, DNA methylation is commonly found at the cytosine of CpG dinucleotide sites in DNA upon replication and is particularly common at gene promoter regions^14,15^. Methylation at a gene promoter region prevents the binding of RNA polymerases and/or other diverse transcriptional factors to the promoter region, thereby inhibiting DNA transcription^16^. Therefore, it is possible to elicit gene expression by demethylating the methylated CpG sites along the promoter region. It is reasonable to assume that a heavily methylated and tightly repressed gene could be a good target for a site-specific demethylation regulating system which would help in understanding the role of DNA methylation. To demethylate CpG sites, the most widely used method has been to inactivate the DNA methyltransferase function through the use of inhibitors including 5-azacytridine (azacitidine) and 5-aza-2-deoxycytidne (decitabine)^17,18^. However, such methods are not gene-specific, resulting in an averaged and thus flattened demethylation effect for global genes. For this reason, these methods are unsuitable for precise targeting of a particular methylated CpG site and its gene regulation.

Recently, investigators have successfully developed efficient epigenetic engineering tools using the CRISPR/Cas9 system which originated from a bacterial immune system that eliminates the genetic materials of invading virues^19–21^. The target DNA, which is a matched specific guide RNA (gRNA) carrying Cas9 based on sequence complementarity, is cleaved into a double strand break (DSB)^19^. The DSB can be repaired by non-homologous end joining (NHEJ)^22^, resulting in insertions and/or deletions (indels) mutation which disrupt the translational reading frame of a coding sequence. Alternatively, if an exogenous DNA, a ‘donor template’ with homology to the target locus, is supplied, the DSB may be repaired by the homology-directed repair (HDR) pathway enabling the insertion of desired sequences^23,24^.

To use the CRISPR/Cas9 system in an epigenetic study, we developed two strategies to regulate the demethylation of the Oct4 promoter and to induce the expression of the Oct4 gene in differentiated cells. Oct4 expressed only in stem cells has been known to be responsible for self-renewal and to maintain the pluripotent state of stem cells^25,26^. Whereas the hypomethylated promoter region of the Oct4 gene in the stem cells is related to its expression, a hypermethylation state in differentiated cells leads to a complete lockdown of Oct4^27,28^.

First, we engineered cells genetically in which the CpG dinucleotides of the promoter region were altered to non-methylated dinucleotides using the CRISPR/Cas9-mediated knock-in strategy. Second, to avoid unknown side-effects caused by the mutated sequence on the promoter, we applied a CRISPR/dCas9-based demethylation system^29^ and made an effort to improve this system by inducing chromatin remodeling. To this end, we used a catalytically inactivated dCas9 fused to the catalytic domain of Ten-Eleven Translocation dioxygenase 1 (Tet1) which catalyzes the conversion of 5-methylcytosine (5-mC) to 5-hydroxymethylcytosine (5-hmC)^30,31^. We examined the effects of the selective demethylation using this dCas9-Tet1 system targeting the methylated CpG sites of the Oct4 promoter along with the synergetic effects of altered histone modifications and DNA methylation. The aim of this research was to investigate a potential selective demethylation tool and especially, to explore new means of epigenetic modulation, and its possible applications in editing hypermethylated genes for clinical purposes.

## Results

### Generation of the CRISPR/Cas9-mediated knock-in system targeting the promoter of Oct4

**Figure 1:**
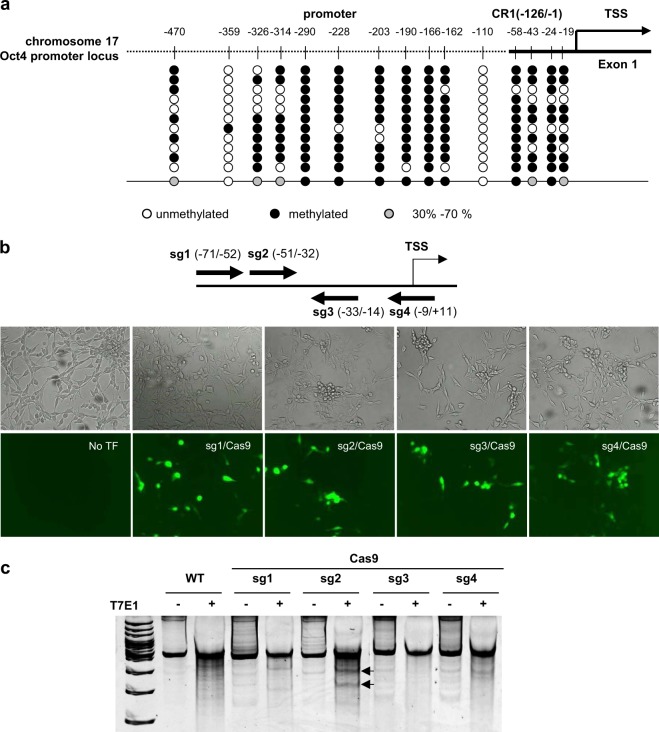
Establishment of the CRISPR system targeting the hypermethylated mouse Oct4 promoter. (**a**) The CpG dinucleotides in the promoter region of the mouse Oct4 gene are shown schematically. The frequencies of methylation at each site in the Oct4 promoter region, determined by bisulfite sequencing, are represented by the open circles, closed circles or gray circles. The horizontal line represents the sequencing result of one clone, and the vertical line represents each individual CpG sites. The open and closed circles represent unmethylated and methylated CpG sites, respectively. The gray circles indicate CpG dinucleotides methylated at 30 to 70 percent frequency. (**b**) Each sgRNA/Cas9-EGFP dual expression vector was transfected into NIH3T3 cells using the microporator, and the expression of Cas9 was confirmed by the fluorescence of GFP. (**c**) Indel efficiency of each sgRNA was confirmed by T7E1 assay. The expected positions of the DNA bands cleaved by T7E1 are indicated by arrows.

**Figure 2:**
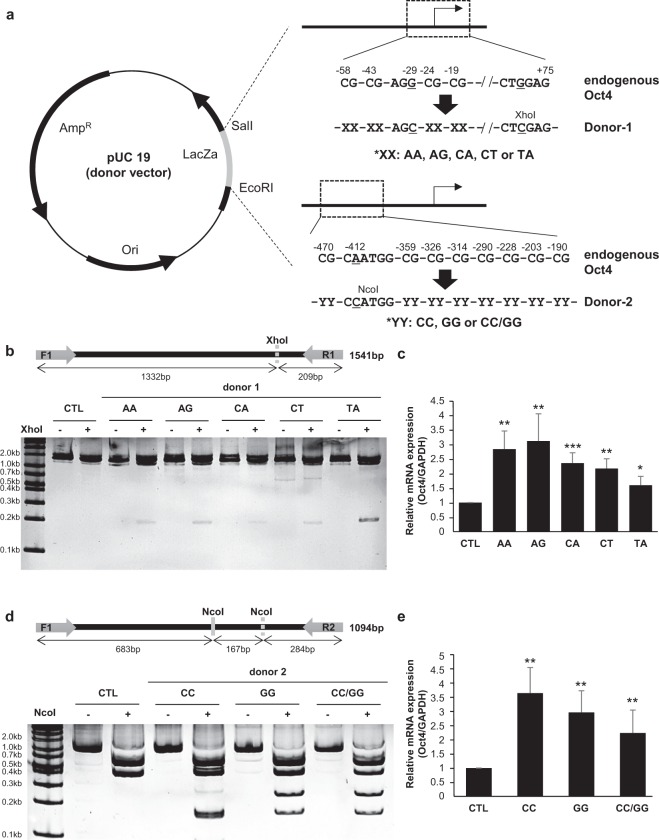
Small-scale replacement of the CpG sites in the promoter region increased the expression of Oct4. (**a**) Schematic of donor DNA constructs targeting the Oct4 promoter is shown. Each donor-1 targeting CR1 among the promoter region contains AA, AG, CA, CT, or TA instead of CpG dinucleotides. PAM sequence for sgRNA2/Cas9 was replaced by AGC, and a XhoI site was newly introduced in donor-1. Donor-2 targeting the rest of the promoter region has CC, GG, or CC/GG, respectively, and a new NcoI site was introduced in donor-2 as a reporter. (**b**,**d**) Genomic DNA was extracted from KI cells in which each donor construct was integrated, and the target region was amplified by PCR. After PCR products were incubated with XhoI (**b**) or NcoI (**d**), respectively, for 1 h, the reaction mixture was separated in 10% polyacrylamide gel. The newly introduced XhoI site and NcoI site in donors are indicated by dotted lines in the upper schematic. (**c**,**e**) mRNA levels of Oct4 from the indicated KI cells were measured by qRT-PCR. The Oct4 mRNA level was normalized to that of GAPDH. Data are presented as the mean ± standard deviation from at least three independent experiments. Statistically significant differences were determined by a two-tailed Student’s *t*-test (*P < 0.05, **P < 0.01, ***P < 0.001).

**Figure 3:**
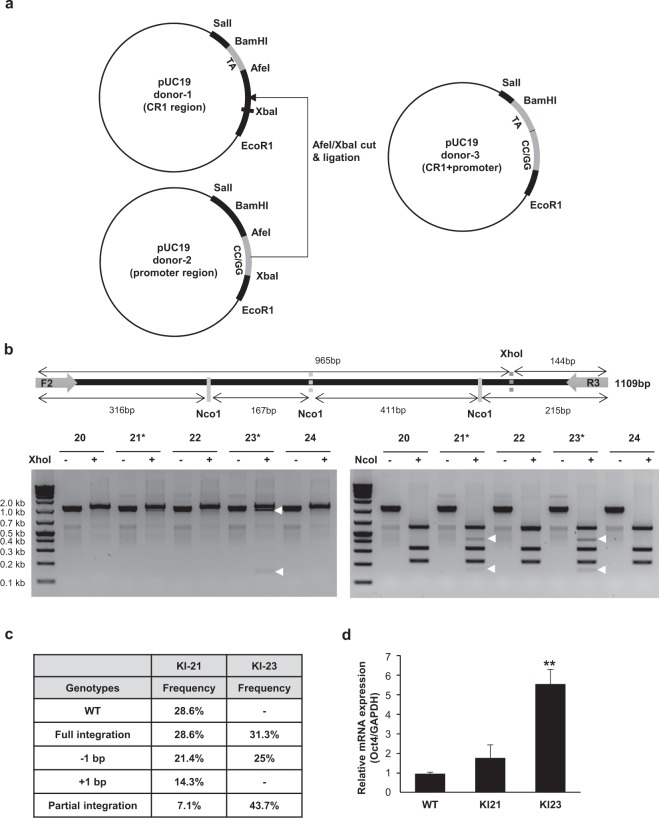
Large-scale replacement of the CpG sites in the promoter region increased the expression of Oct4. (**a**) A scheme for producing donor-3 targeting the entire promoter of Oct4 is illustrated. DNA fragment from donor-2 treated by AfeI/XbaI was ligated to donor-1 vector which was cut by AfeI/XbaI. (**b**) The newly introduced XhoI site and NcoI site in donor-3 are indicated by dotted lines in the upper schematic. PCR products from isolated KI colonies were treated by XhoI or NcoI, respectively, and then, the reaction mixture was separated on a 2% agarose gel. The expected positions of the DNA bands cleaved by XhoI and Nco1 are indicated by white arrows. (**c**) The genotypes of selected cell lines were analyzed by Sanger sequencing, and the frequency of each genotype was indicated. (**d**) mRNA levels of Oct4 from the indicated cells were measured by qRT-PCR. The Oct4 mRNA level was normalized to that of GAPDH. Data are presented as the mean ± standard deviation from at least three independent experiments. Statistically significant differences were determined by a two-tailed Student’s *t*-test (**P < 0.01).

First, we investigated the DNA methylation profile of the Oct4 promoter region up to −470 bp from the transcription start site (TSS) in the NIH3T3 cells using bisulfite sequencing. There are 15 CpG dinucleotides in this region including conservative region 1 (CR1) (−126 to −1 site)^32^, and the overall percentage of CpG methylation was about 69.1% (Fig. 1a). In the NCBI database (NCBI Reference Sequence: NC_000083.6), it is known that there are 16 CpG sites in this region; however, in the NIH3T3 cells used for our experiments, the cytosine at the −420 site was substituted with thymine, resulting in a total of 15 CpGs (Supple. Fig. 1). Of these, the cytosine at the −110 site was thoroughly unmethylated as reported in a previous study^27^; thus, this site was excluded from our target list. First, we divided the target into the CR1 region and the upstream promoter region because four CpG sites in CR1 are critical for the regulation of the Oct4 gene expression. Those sites were also heavily methylated exhibiting 75% methylation (Fig. 1a and Supple. Fig. 1). It is well known that the Oct4 gene expression is mostly regulated by DNA methylation in the promoter region^27,28^. Therefore, we attempted to regulate the Oct4 gene expression by the site-specific regulation of DNA methylation of this region. The first approach was to obviate DNA methylation by replacing the CpG sites with non-DNA methylation consensus sequences using homology-directed recombination (HDR)-mediated gene correction. To this end, we established a CRISPR/Cas9 system with four individual single guide RNAs (sgRNAs), each of which target the individual CpG sites in the CR1 region. SpCas9 and sgRNA were overexpressed by transfecting the sgRNA/Cas9 dual expression vector constructs into NIH3T3 cells (Fig. 1b). Then, we assessed the indel efficiency for each target using a T7E1 assay. Among those four targets, sgRNA-2 showed the highest indel efficiency (Fig. 1c). Next, five different donor plasmid DNAs were constructed that carried non-methylated dinucleotide sequences (AA, AG, CA, CT, or TA) and homology arms with a length of 1600 bp. These plasmids were used as donor templates for HDR-mediated gene correction (Fig. 2a). The PAM sequence (AGG to AGC) of each donor plasmid was also modified to avoid cleavage of the integrated donor by sgRNA2/Cas9. We also included a XhoI restriction site (CTGGAG to CTCGAG) in the donor plasmid as a reporter sequence (Fig. 2a and Table 1). Donor plasmids targeting the upstream promoter region were also designed to substitute the CpG nucleotides with either CC, GG, or CC/GG dinucleotides. An NcoI restriction site (CCATGG) was also included (Fig. 2a and Table 1). Particularly, the CC/GG donor DNA was designed to avoid the occurrence of any newly formed CG dinucleotides (Fig. 2a and Table 1). For example, when ‘CCG (−470)’ is changed to ‘CGG’, a new CG site is newly created one-base upstream (–471). However, we did not perform a sequence substitution at the −166 and −161 sites which overlap with the AfeI enzyme site (Fig. 3a and Supple. Fig. 1). Each of the constructed donor plasmids was transfected into NIH3T3 cells together with a sgRNA2/Cas9 expression vector. Then, sequence-substituted clones were isolated that showed DNA fragments on acrylamide gels following treatment of the PCR products with the XhoI or NcoI restriction enzymes (Fig. 2b,d). The sequence substitutions were confirmed by Sanger sequencing (Supple. Fig. 2). The sequencing results indicated that each clone showed substitutions at one to three alleles among the 4 alleles in the NIH3T3 cells.

**Table 1:** Engineered sequences on donor DNA.

CpG position		−58 −43 • −24 −19 *
Wild type DNA sequence		AC**CG**TCCCTAGGTGAGC**CG**TCTTTCCACC**AGG**CCCC**CG**GCT**CG**GG--------AGGG**CTGGAG**CCGGG
Donor DNA	AA	AC**AA**TCCCTAGGTGAGC**AA**TCTTTCCACC**AGC**CCCC**AA**GCT**AA**GG--------AGGG**CTCGAG**CCGGG
	AG	AC**AG**TCCCTAGGTGAGC**AG**TCTTTCCACC**AGC**CCCC**AG**GCT**AG**GG--------AGGG**CTCGAG**CCGGG
	CA	AC**CA**TCCCTAGGTGAGC**CA**TCTTTCCACC**AGC**CCCC**CA**GCT**CA**GG--------AGGG**CTCGAG**CCGGG
	CT	AC**CT**TCCCTAGGTGAGC**CT**TCTTTCCACC**AGC**CCCC**CT**GCT**CT**GG--------AGGG**CTCGAG**CCGGG
	TA	AC**TA** TCCCTAGGTGAGC**TA**TCTTTCCACC**AGC**CCCC**TA**GCT**TA**GG--------AGGG**CTCGAG**CCGGG
CpG position		−470 * −359 −326 −314 −290 −228 −203 −190
Wild type DNA sequence		A**CG**G----- T**CAATGG**A------C**CG**A---G**CG**A--C**CG**A--C**CG**G---C**CG**T----A**CG**T---C**CG**T
Donor DNA	CC	A**CC**G----- T**CCATGG**A------C**CC**A---G**CC**A--C**CC**A--C**CC**G---C**CC**T----A**CC**T---C**CC**T
	GG	A**GG**G----- T**CCATGG**A------C**GG**A---G**GG**A--C**GG**A--C**GG**G---C**GG**T----A**GG**T---C**GG**T
	CC/GG	A**GG**G----- T**CCATGG**A------C**CC**A---G**GG**A--C**CC**A--C**CC**G---C**CG**T----A**GG**T---C**CC**T

^●^The site is the PAM sequence for the sgRNA2/Cas9. *The sites are modified for the introduction of the restriction enzyme site as a reporter.

### Transcriptional regulation of Oct4 gene expression via HDR-mediated gene correction

To gauge the dependency of the Oct4 gene expression on CpG DNA methylation in CR1, the transcription levels of Oct4 in each cloned cell were measured by a quantitative RT-PCR method. The results indicated that the HDR-mediated demethylation led to an 1.5- to 3-fold increase in the Oct4 mRNA level depending on the mutated dinucleotide sequences (Fig. 2c). In particular, the up-regulation of Oct4 gene transcription was most pronounced in cells with an ‘AG’ dinucleotide mutation. Independent of the CR1 mutations, approximately a 2-4 fold increase in Oct4 gene transcription was observed in cell clones with dinucleotide substitutions in the upper promoter region (Fig. 2e). In this case, the activation of Oct4 gene transcription was more pronounced in the ‘CC’ dinucleotide substitution (Fig. 2e). From these results, we provided tools to activate the quiescent gene expressions through programmable nuclease-triggered, HDR-mediated gene corrections, in which ‘CG’ was substituted with non-methylated dinucleotides. The ‘AG’ substitution showed more pronounced effects on gene activation, but it awaits further scrutiny whether the dinucleotide sequence can be universally applicable for universal ‘CpG’ sequences, or the optimal sequence is target-dependent.

### Combinatorial alteration of the CpG sites in CR1 and the upper promoter region

To replace the CpGs over a wider range of the promoter region, we used the established donors to create a new donor that had replaced the CpGs in both the CR1 and the rest of the promoter region. The donor with the TA dinucleotides (donor-1) and the donor with the CC/GG dinucleotides (donor-2) were digested with AfeI/XbaI, and the digested CC/GG donor fragment was ligated into the TA donor vector (Fig. 3a). The TA donor was chosen because it showed the highest KI efficiency in the previous experiments, and the CC/GG donor was selected to avoid the generation of new CpGs. In the previous results, the substitution rate of the non-methylated dinucleotides and the amounts of Oct4 expression were not directly proportional. The TA donor was integrated into 3 of the 4 alleles, and the AG donor was integrated into only one allele, but Oct4 expression was dominant in the colony cells carrying AG dinucleotides. These results are believed to be due to other factors not related to DNA methylation. The new donor (donor-3) was transfected into NIH3T3 cells together with the sgRNA2/Cas9 plasmid DNA, and puromycin-resistant single-cell colonies were isolated. Genomic DNA was extracted from these colonies, and the target region was amplified by PCR, followed by digestion with XhoI and NcoI, respectively. From this screening, the 23^rd^ colony (KI-23) was revealed to contain both selectable markers (Fig. 3b). The PCR product from the 21^st^ colony (KI-21) also showed the DNA fragments at the expected sizes with the NcoI digestion, although the cleavage by XhoI was only weakly observed. Sequencing analysis of these colony cells verified that the KI-23 cells have three genotypes, but the KI-21 cells have five genotypes (Fig. 3c). Considering that NIH3T3 cells have four 17-chromosomes^33^, the KI-21 cells seem to contain two types of cells. Interestingly, it is certain that the KI-23 cells originate from a single cell, but two of the four 17-chromosomes contain partially integrated donor DNA and only one of the four chromosomes was replaced as we designed (Supple. Fig. 3). This partial HDR has already been reported in other studies^32,34^ and is believed to be due to the complicated DNA repair process. However, we decided to compare the Oct4 transcription level in both these cells because they showed different substitution rates. The Oct4 mRNA level of the KI-23 cells was 5.6-fold higher than that of the wild-type NIH3T3 cells, while the Oct4 mRNA level of the KI-21 cells was about 1.8-fold higher. This may be because the KI efficiency of the KI-23 cells (including the full and partial integration) reached 75% while that of the KI-21 cells was only 35%.

### Effects of targeted demethylation using dCas9-Tet1 modules on Oct4 gene activation

**Figure 4:**
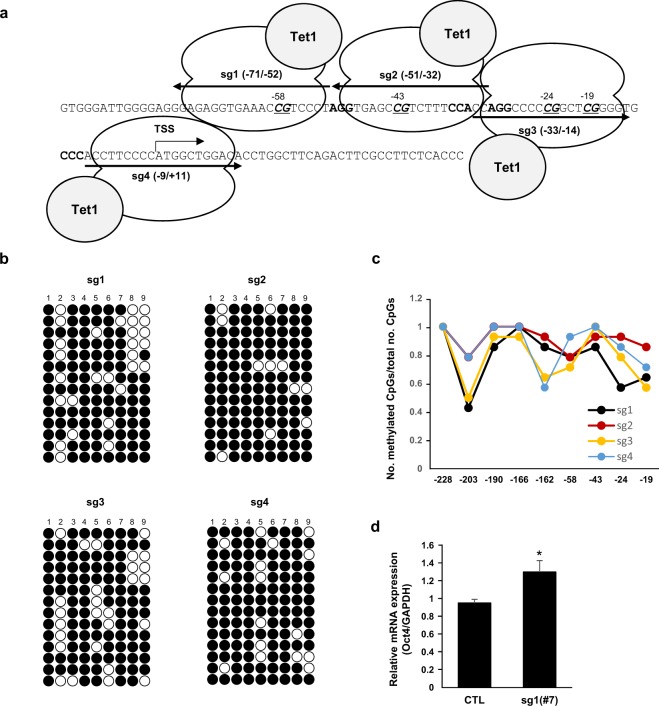
sgRNA/dCas9-Tet1 system was designed for the demethylation of the Oct4 promoter. (**a**) Schematic for the sgRNA/dCas9-Tet1 modules targeting the Oct4 promoter. Italic and bold letters indicate the CpG sites and PAM sequences for each module, respectively. Corresponding sequences for each sgRNA were identical to Fig. 1. (**b**) The sequence −228 to −19 from TSS of the Oct4 promoter was analyzed by bisulfite sequencing. Fourteen clones per sample were sequenced and the sequence results are shown in the dot plot. The horizontal line represents the sequencing result of one clone, and the vertical line represents each individual CpG sites. The numbers (1–9) on top indicate the −228, −203, −190, −166, −162, −58, −43, −24, and −19 sites sequentially. The open and closed circles represent unmethylated and methylated CpG sites, respectively. (**c**) The graphs show the rate of methylated CpGs (y-axis) for the different sites along the Oct4 promoter (x-axis) based on the result of b. (**d**) mRNA levels of Oct4 from NIH3T3 cells and sgRNA1/dCas9-Tet1 stable expressing NIH3T3 cells were measured by qRT-PCR. The Oct4 mRNA level was normalized to that of GAPDH. Data are presented as the mean ± standard deviation from at least three independent experiments. Statistically significant differences were determined by a two-tailed Student’s *t*-test (*P < 0.05).

The HDR-mediated gene correction strategy should include the isolation step of substituted clones, and this procedure may be applicable to *ex vivo* gene therapy in which induced pluripotent stem cells or hematopoietic stem cells are subjected to gene correction, and then, gene-corrected clones are introduced into patients. However, this strategy poses significant hurdles because HDR efficiency is not yet satisfactory. Thus, we tested whether targeted demethylation using the dCas9-Tet1 system^29,31,35^ could provide an alternative method to the HDR-mediated gene correction strategy. To this end, we constructed a catalytically inactive Cas9 (dCas9) fused to the catalytic domain of Tet1, an enzyme involved in DNA demethylation. This method has already been tested in several cells and found to be effective in regulating gene expressions without introducing alterations to the genome. To test whether the up-regulation of Oct4 gene expression is indeed dependent on the site-specific DNA methylation, we targeted the dCas9-Tet1 system using four sgRNAs designed to target CpG sites in the CR1 region and then measured the methylation state of the CpGs at the promoter (−228 to −19 site) through bisulfite sequencing (Fig. 4a). The overall percentages of CpG methylation of sgRNA1- and sgRNA3-transfecting cells were 78% and 79% respectively (Fig. 4b,c), whereas that of wild-type NIH3T3 cells was 82% in the same region (Fig. 1a). Although the demethylation effects were not exclusively site-specific, sgRNA1 and sgRNA3 showed the demethylation effects at −203 and −24 sites, and at −203 and −162 sites respectively (Fig. 4b,c). Despite the confirmed site-specific demethylation at several CpG sites, the demethylation did not culminate in a significant degree of Oct4 gene activation (data not shown). To confirm the long-term effects of dCas9-Tet1, we established a stable cell line expressing sgRNA1/dCas9-Tet1 because sgRNA1 showed the most prominent demethylation effects (Supple. Fig. 4). However, significant increase of Oct4 mRNA was not also observed in a cell clone with a stable overexpression of sgRNA1/dCas9-Tet1 (Fig. 4d). This result urged us to adopt an additional strategy to complement the marginal effects of the dCas9-Tet1 modules on gene activation.

### Synergetic effects of altered histone modifications and DNA methylation on Oct4 expression

**Figure 5:**
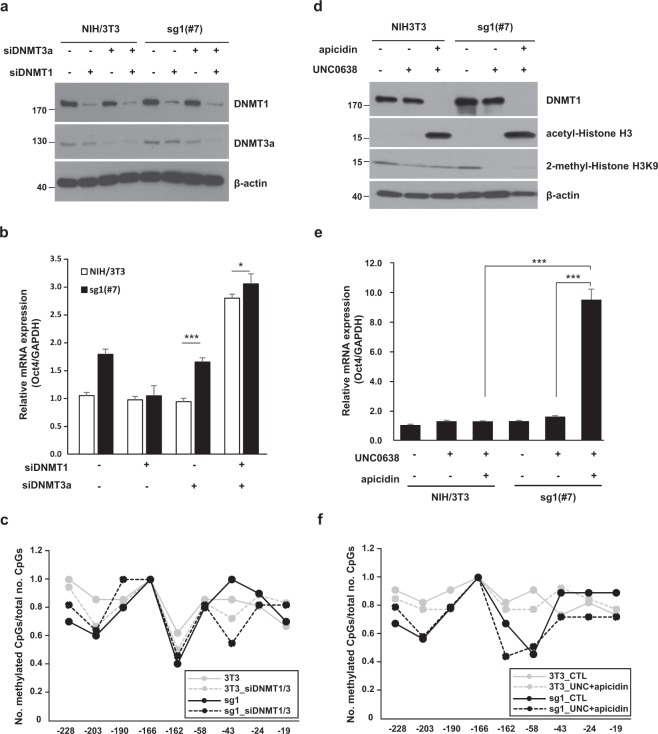
Combination of histone modifications and DNA methylation showed a substantial synergetic effect on the Oct4 expression. (**a**) Endogenous DNMT-1 and DNMT-3a in NIH3T3 cells and sgRNA1/dCas9-Tet1 stable expressing NIH3T3 cells were knocked down by siRNA. The lysates were immunoblotted with the relevant antibodies described in the “Materials and Methods”. Full-length blots are presented in Supplementary Fig. 5. (**b**,**e**) mRNA levels of Oct4 from the indicated cells were measured by qRT-PCR. The Oct4 mRNA level was normalized to that of GAPDH. Data are presented as the mean ± standard deviation from at least three independent experiments. Statistically significant differences were determined by a two-tailed Student’s *t*-test (*P < 0.05, ***P < 0.001). (**d**) NIH3T3 cells and sgRNA1/dCas9-Tet1 stable expressing NIH3T3 cells were treated with 2 μM apicidin (for 48 h) and/or 1 μM UNC0638 (for 96 h), and the cell lysates were immunoblotted with the relevant antibodies described in the “Materials and Methods”. Full-length blots are presented in Supplementary Fig. 5. (**c**,**f**) The graphs show the rate of methylated CpGs (y-axis) for the different sites along the Oct4 promoter (x-axis). Methylation statuses under the indicated conditions were examined using bisulfite sequencing. The original bisulfite sequencing results are presented in Supplementary Fig. 6.

To improve the demethylation effect of the dCas9-Tet1 system, we transfected DNA methyltransferase (DNMT) 1- and/or DNMT3a-specific small interfering RNA (siRNA) into the sgRNA1/dCas9-Tet1 stable cell lines (Fig. 5a). Western blot analysis revealed that the DNMT1- and DNMT3a-siRNA treatment efficiently suppressed the corresponding protein expressions (Fig. 5a). In this condition, levels of Oct4 mRNA were increased by approximately 3-fold in both NIH3T3 cells and sgRNA1/dCas9-Tet1 stable cell lines (Fig. 5b). Thus, it is estimated that this increment is the result of DNMT gene silencing, independently of sgRNA1/dCas9-Tet1 overexpression. The demethylation effects by the DNMT gene silencing were not detected except for −43 sites in both cells (Fig. 5c). Silencing of DNMT genes has a potential for genome-wide effect on the methylation pattern, but this effect was confined to the specific site of Oct4 promoter in our results. Nonetheless, even the complementary treatment of siRNAs was not sufficient in Oct4 gene activation. We then attempted to further boost Oct4 gene activation by chromatin remodeling. We treated NIH3T3 cells with a histone deacetylase inhibitor (apicidin) and/or a histone methyltransferase inhibitor (UNC0638). We inferred that this treatment would help the sgRNA1/dCas9-Tet1 ribonucleoprotein complex access the otherwise compact promoter region. In general, transcriptionally quiescent genes are more probable to form a heterochromatin structure. Acetylation and methylation status of histones in the heterochromatin region have an important role in maintaining the aggregated structure to inhibit gene expression^36^. Our hypothesis could be reasonable based on the finding that Cas9 protein more readily accesses disengaged DNA from the histone octamer by chromatin remodeling enzymes^37^. The effects of these inhibitors on the histone modifications was confirmed by Western blot (Fig. 5d). Interestingly enough, the expression of DNMT1 in the apicidin-treated cells was completely blocked; this phenomenon has been reported in a previous study^38^. The treatment of apicidin resulted in the acetylation of histone 3 as well as the suppression of DNMT1 expression, but the methylation of histone H3K9 by UNC0638 decreased slightly. These chromatin remodeling induced a significant up-regulation of Oct4 gene expression by approximately 10-fold in the sgRNA1/dCas9-Tet1 stable cell line (Fig. 5e). It is important to note that the gene activation was exclusively observed in the presence of sgRNA1/dCas9-Tet1 modules. In this case, it was also confirmed that demethylation in the CR1 region including −43 site was significantly increased in sgRNA1/dCas9-Tet1 stable cell line (Fig. 5f). The observed demethylation effect might be because of a combination of the easy accessibility of dCas9 by chromatin remodeling and the silencing effect of DNMT1 by apicidin. Taken together, we concluded that the dCas9-Tet1 system can effectively activate a transcriptionally quiescent gene via targeted DNA demethylation, when supplemented by both histone H3 acetylating- and Histone H3K9 demethylating-agents.

## Discussion

The importance of epigenetic research has been emerging because it provides clues on gene regulation. Especially, there have been many attempts to modulate gene expression by manipulating DNA methylation. However, despite the many studies, it has been difficult to modify the methylation of DNA selectively. In conventional demethylation methods, several inhibitors of DNMTs have been widely used, but they are not suitable for modulating a specific gene due to the lack of their specificity. For these reasons, the development of methods to modify DNA methylation selectively is important in gene regulation and further applications in therapy. This study was designed to develop a new method to edit DNA methylation selectively and to induce gene expression.

The recent advent of genome editing technologies like CRISPR/Cas9 has facilitated selective targeting to specific genomic locus using guide RNA. According to previous studies, CRISPR/Cas9-induced HDR-mediated knock-in was able to edit specific DNA sequences *in vitro* and *in vivo*^39,40^. In addition, dCas9 and various fusions of sgRNA/dCas9 with a transcriptional modulator have been used to regulate gene expression at targeted loci without introducing alterations to the genome^41–43^. Recently, a case has been reported in which human somatic cells were reprogrammed into iPSCs by simultaneously targeting various endogenous genes using CRISPR activation (CRISPRa) tools^44^. Based on these findings, we induced site-specific demethylation in the Oct4 promoter region with the CRISPR/Cas9 system and elicited Oct4 gene expression.

Using bisulfite sequencing, we revealed the state of the CpG methylation in the promoter of the Oct4 gene in differentiated NIH3T3 cell. This result shows similarities to previous reports that most CpG islands were hypermethylated especially the four CpG sites of CR1 near the transcription start site. In this study, we edited the promoter region of Oct4 by HDR-mediated gene correction, thereby preventing methylation of the edited region. We established bioengineered cell lines in which the CpG dinucleotides of the promoter region were altered to another dinucleotide. As a result, Oct4 expression of all the engineered NIH3T3 cells was increased compared with that of the wild-type NIH3T3 cells. In particular, the increase in Oct4 expression was the greatest in cells that had the CpG dinucleotides altered in the entire promoter region which included the CR1 region.

Studies on the transcription factor of the CR1 region in the Oct4 promoter have been well reported^45,46^. According to the reports, the transcription factor binding sites in CR1 are as follows: a putative Sp1/Sp3 binding site (−115/−106), a steroidogenic factor 1 (SF1 or Nr5a1) binding site (−101/−70), a 1A-like site (−77/−70), and a hormone-responsive element (HRE) site (−108/−103, −101/−96, and −95/−90). Fortunately, our sequence substitutions did not fall on those sites. Nonetheless, we could not rule out the possibility of the existence of any potential transcription factor binding site. That’s why we supplemented our approach with, a second strategy using dCas9-Tet1 system. Previous studies have shown that the dCas9-Tet1 system induces site-specific demethylation^47–49^. Based on these findings, we used the dCas9-Tet1 module targeting the Oct4 promoter without any alteration of the DNA sequence and induced demethylation selectively. However, the effects of the demethylation using the dCas9-Tet1 system was temporary and unremarkable. We tried to induce site-specific demethylation using different types of guide RNAs but had no actual results. Each demethylation efficiency was also different, and site-specific demethylation effects could not be asserted. The different effects of each sgRNA/dCas9-Tet1 may be due to not only the complicated nucleotide-chromatin three-dimensional structure but also the distribution of various transcriptosomes. In addition, despite establishing a stable cell line that expresses sgRNA1/dCas9-Tet1, there was little effect on Oct4 expression. Demethylation, which we induced, may not have met the necessary and sufficient conditions for Oct4 expression. However, when the sgRNA1/dCas9-Tet1 expressing NIH3T3 cells were co-treated with histone deacetylase and histone methyltransferase inhibitors, the expression of the Oct4 gene was remarkably induced. It was shown that DNA demethylation and histone modification synergistically affect the Oct4 expression. Some previous studies have mainly focused on the effect of DNA methylation in repressing Oct4 expression^50,51^, but based on our results, Oct4 expression is tightly regulated not only by methylation but also by histone modifications in the promoter. In addition, it is widely accepted that the expression of Oct4 is induced by the complimentary action of histone modification and DNA methylation because the major transcription factors recruited to the Oct4 promoter are well known to be involved in histone modification^46^.

In conclusion, we introduced two methods to regulate DNA methylation selectively using the CRISPR/Cas9 system and successfully induced gene expression. However, we encountered several problems in applying this HDR-mediated gene correction strategy; one is the low KI efficiency, and the other is the partial-HDR. These are common problems in the KI strategy using the CRISPR system, and therefore, in addition to a method that maximizes the KI efficiency, designing a donor that takes into consideration the partial HDR should be done concurrently. We suggest that a HDR-mediated gene correction strategy is suitable for inducing site-specific mutations in one or adjacent sites. Overcoming these limitations will promise broader use of HDR-mediated gene correction, in particular in *ex vivo* gene therapy. In addition, although methods for controlling DNA methylation using dCas9-Tet1 have already been introduced, we suggest that the addition of other epigenetic modifiers can further increase the efficiency of this system. It is not easy to identify the precise point of action of the dCas9-epigenetic modulator because the tertiary structure of nucleosome-chromatin is very complicated and dynamic according to cellular events. Therefore, the correlation between epigenetic factors as well as a precise structural analysis of the nucleosome-chromatin should be further investigated. It is certain that if the regulation method of DNA methylation is further refined based on our suggestion, this can be used extensively in various fields.

## Materials and Methods

### Plasmids construction

For targeting mouse Oct4 and inducing the DSB on the target region, the single guide RNA (sgRNA) coding sequence was incorporated into the BbsI (R0539S, New England Biolabs) site of the bicistronic expression plasmids PX458 or PX459, respectively^19^ (#48138, #48139, Addgene). Pairs of DNA oligonucleotides harboring variable 24 nucleotide sequences were annealed and ligated into the PX458 or PX459 plasmids as previously described^19^. The corresponding binding sites of the four sgRNA and their locations are described in Supple. Table S1. Donor constructs for genome editing were generated consisting of upper and lower arms (1600 bp) homologous to the targeted cut site. Homology arms were PCR amplified from genomic DNA extracted from NIH3T3 (ATCC, USA) cells and cloned into EcoRI (R0101S, New England Biolabs) and SalI (R0138S, New England Biolabs) digested pUC19 vector (EZ002S, Enzynomics). Then, synthesized a 260-mer oligonucleotide containing altered CpG dinucleotides and reporter sites was cloned into the above donor pUC19 vector following digestion with AfeI (R0652L, New England Biolabs) and BamHI (R0136S, New England Biolabs). The dCas9-Tet1 plasmid was constructed based on the parental plasmid PX458. Two nuclease activity sites of Cas9 were mutated by the site-directed mutagenesis method (D10A, H840A). Next, EGFP gene was deleted by EcoRI (R0101S, New England Biolabs) restriction enzyme digestion, and then, the Tet1 catalytic domain (Tet1-CD) was fused to dCas9. All constructs were verified by Sanger sequencing prior to use.

### Cell culture and treatment

NIH3T3 cells were cultured in Dulbecco’s modified Eagle’s medium (Gibco, USA) supplemented with 10% fetal bovine serum (Gibco, USA) and 1% penicillin/streptomycin (Gibco, USA). Cells were incubated at 37 °C in a humidified 5% CO_2_ incubator. Cells were treated with 1 μM UNC0638 (Sigma, USA) or 2 μM apicidin (Sigma, USA) for 96 or 48 h, respectively, and compound-containing media was refreshed every 24 h.

### Transfection

To transfect the various plasmids including sgRNA/Cas9, donors, and sgRNA/dCas9-Tet1 into NIH3T3, the Microporator (Digital Bio, Korea) was used. Microporation was performed using the manual’s recommended program: 1680 voltage, 15 ms, and two pulses. Cells transfected with each of 4 different types of sgRNA/dCas9-Tet1 were harvested 72 h later to examine the DNA demethylation pattern.

To isolate the knock-in cell lines, cells were initially transfected with the sgRNA/Cas9 plasmid including puromycin resistant gene (PX459) and donor DNA and subjected to screening for puromycin resistance to obtain cells that have altered CpG sites on the Oct4 promoter. Colonies were picked and harvested for RNA and genomic DNA extraction to examine the expression of Oct4 and the efficiency of the knock-in. The primer sequences used for amplifying the target region were as follows:

Primer F1: 5′-TCTAGGGAAGTTCAGGGTAGG-3′

Primer F2: 5′-CCCCTTTGAACCTGAAGTC-3′

Primer R1: 5′-CCTGGCCCTCAGGCTGCAAA-3′

Primer R2: 5′-TATCTGCCTGTGTCTTCCA-3′

Primer R3: 5′-TCCACAGTATGCCATCCCTC-3′

To establish stable cell line overexpressing sgRNA1/dCas9-Tet1, sgRNA1/dCas9-Tet1 plasmid (PX459) was transfected to NIH3T3 cells and subjected to primary screening of puromycin-resistant cells. Proper integration and stable expression of sgRNA1/dCas9-Tet1 gene in the selected colonies were confirmed by PCR, Sanger sequencing, and Western blot (Supple. Fig. 4).

siRNA transfection of NIH3T3 cells was performed using Lipofectamine RNAi MAX (Invitrogen, USA) according to the manufacturer’s transfection protocol. The siRNA sequences used in this study were as follows:

siDNMT1 sense: 5′-CAGAUAGCUACCGGUUCUU (dTdT)-3′

siDNMT1 anti-sense: 5′-AAGAACCGGUAGCUAUCUG (dTdT)-3′

siDNMT3a sense: 5′-CUGUGUACGUGAUGCUUGU (dTdT)-3′

siDNMT3a antisense: 5′-ACAAGCAUCACGUACACAG (dTdT)-3′

### T7E1 assay

To verify the indel mutations, the target region was amplified using the following primers: Oct4 sequencing forward primer (5′-GGTTGAAAATGAAGGCCTCCTG-3′) and Oct4 sequencing reverse primer (5′-CCTGGCCCTCAGGCTGCA AA-3′). PCR products were incubated with T7E1 for 30 min at 37 °C. The reaction products were analyzed by electrophoresis with a 10% polyacrylamide gel or 2% agarose gel.

### Quantitative real-time PCR (Oct4 expression analysis by qPCR)

Total RNAs were extracted from the cells of the indicated groups using a RNA Extraction kit (Nanohelix, Korea) and converted to the complementary DNA using the 1^st^ cDNA synthesis kit (Nanohelix, Korea). The change in fold expression of Oct4 was then determined with a SYBR green master mix (Thermo, USA), using the QuantStudio 3 real-time PCR system (Thermo, USA). The relative abundances of the transcripts of Oct4 gene were normalized to that of the GAPDH gene. All data were obtained from at least three independent experiments. Primer sequences for the Oct4 and GAPDH were as follows:

Oct4 forward: 5′- GCCCTCCCTACAGCAGATCACTCACATCG-3′

Oct4 reverse: 5′-AAGGTGTCCCTGTAGCCTCATACTCTTCTCGT-3′

GADPH forward: 5′- GGGCATCTTGGGCTACACTGAGGACCAG-3′

GAPDH reverse: 5′- CACCCTGTTGCTGTAGCCGTATTCATTGTC-3′

### Bisulfite DNA sequencing

Genomic DNA was extracted from the cells of the indicated groups using the Genomic DNA extraction kit (Nanohelix, Korea). Genomic DNA was treated with bisulfite using the Bisulfite conversion system (Promega) according to the manufacturer’s instruction. The converted DNA was amplified by Epitaq (Takara, Japan) using the following primers: Oct4 bisulfite forward primer (5′-GTTTTGGATATGGGTTGAAATATTG-3′) and Oct4 bisulfite reverse primer (5′-CCCCACCTAATAAAAATAAAAAAAC-3′). Then, the PCR products were cloned into T-blunt vector (Solgent, Korea), followed by Sanger sequencing.

### Western blot

Cells were collected and washed with PBS and lysed in NP40 (Nonidet-P40) buffer supplemented with protease inhibitor cocktails. The protein concentration was measured using the Bradford assay (Bio-Rad, USA). Equal protein amounts were separated on a 12% SDS-PAGE gel by electrophoresis and transferred to a PVDF membrane (EMD Millipore, USA). The membrane was blocked with 5% skimmed milk in TBST at room temperature for 1 h and incubated with primary antibodies overnight at 4 °C. The membranes were then washed with TBST and incubated with secondary antibody for 1 h at room temperature. Signals were detected using the Amersham^TM^ ECL^TM^ Western Blotting Detection Reagents (GE Healthcare, USA) and exposed onto an X-ray film. Antibodies against FLAG (F3165, Sigma), beta-actin (ab8227, Abcam), DNMT1, 3a (#5119, #2160, Cell Signaling Technology), dimethyl-histone H3K9 (ab1220, Abcam), and acetyl-histone H3 (ab47915, Abcam) were used.

### Statistical analysis

Data were expressed as the means ± SD based on at least three independent experiments. Statistical analysis was performed by unpaired t-test for two groups.

## Supplementary information

Supplementary Information
