# Supplementary material for: Regulation of gene expression by altered promoter methylation using a CRISPR/Cas9-mediated epigenetic editing system
Source: Sci Rep. 2019 Aug 19;9:11960. doi: 10.1038/s41598-019-48130-3 (PMC6700181; doi:10.1038/s41598-019-48130-3)

Supplementary information

Regulation of gene expression by altered promoter methylation using a CRISPR/Cas9-mediated epigenetic editing system

Jeong Gu Kang[1], Jin Suk Park[1,2], Jeong-Heon Ko[1,2,*] and Yong-Sam Kim[1,2,*]

[1]Genome Editing Research Center, Korea Research Institute of Bioscience and Biotechnology (KRIBB), 125 Gwahak-ro, Yuseong-gu, Daejeon 34141, Korea

[2]Department of Biomolecular Science, KRIBB School of Bioscience, Korea University of Science and Technology (UST), 217 Gajeong-ro, Yuseong-gu, Daejeon 34113, Korea

*These authors contributed equally: J.G.K and J.S.P

**Correspondence and requests for materials should be addressed to J.-H.K or Y.-S.K. (email: jhko@kribb.re.kr or omsys1@kribb.re.kr)

Supplementary Figure Legends

Supplementary Figure 1. Alignment of the Oct4 promoter sequence. Reference sequence from the NCBI database (NC_000083.6: 35,505,000 – 35,505,575) and sequence result of the genomic DNA extracted from NIH3T3 cells were aligned using GeneDoc DNA sequence analysis tool (Copyright©2006 by Karl Nicholas). The red boxes and the black line indicate the CpG sites and exon region, respectively. Transcription start site (TSS) and AfeI restriction enzyme site are also shown.

Supplementary Figure 2. Validation of the engineered cells by Sanger sequencing. DNA sequence chromatogram shows the proper alterations of the target region. Altered CpG sites and the reporter sequence are underlined by black, and the wild-type sequences are underlined by gray.

Supplementary Figure 3. Genotypes of the KI-23 cells. Genomic DNA extracted from the KI-23 cells and target region was amplified by PCR. TA cloning was performed with the PCR products. Sixteen clones were sequenced by Sanger sequencing and the results were aligned using GeneDoc. Three types of genotypes were presented; a) 1 bp deletion, b) full integration, and c) partial integration. The blue and red boxes indicate the altered CpGs in CR1 and the rest of the promoter, respectively. The red underline indicates the PAM sequence for sgRNA2/Cas9, and the black lines represent the XhoI/NcoI restriction enzyme sites introduced by KI, respectively.

Supplementary Figure 4. Validation of sgRNA1/dCas9-Tet1 stable expressing NIH3T3. (a) Cell lysates from sgRNA1/dCas9-Tet1 stable expressing NIH3T3 cells (colony number: #7, #8, and #10) were immunoblotted with anti-Flag antibody and anti-beta-actin antibody. (b) Genomic DNA was extracted from NIH3T3 cells and sgRNA1/dCas9-Tet1 stable expressing NIH3T3 cells (#7), and PCR was performed using single guide RNA specific primers which are shown in the right schematic. The sgRNA1/dCas9-Tet1 dual expression vector was used as the positive control (P.C.). (c) PCR products were analyzed by Sanger sequencing. (d) The sequence -228 to -19 from TSS of the Oct4 promoter was analyzed by bisulfite sequencing. The horizontal line represents the sequencing result of one clone, and the vertical line represents each individual CpG sites. The numbers (1-9) on top indicate the -228, -203, -190, -166, -162, -58, -43, -24, and -19 sites sequentially. The open and closed circles represent

unmethylated and methylated CpG sites, respectively. The graphs show the rate of methylated CpGs (y-axis) for the different sites along the Oct4 promoter (x-axis) based on the bisulfite sequencing result.

Supplementary Figure 5. Uncropped immunoblots, corresponding to the indicated figures in the manuscript.

Supplementary Figure 6. Original bisulfite sequencing results, corresponding to the indicated figures in the manuscript.

Supplementary Table S1. Guide RNA target sequences for CRISPR system

guide RNA	Target sequence (5‘ - 3‘)	Distance to TSS (bp)
sg1	**GGAGAGGTGAAACCGTCCCT**	-71/-52
sg2	**AGGTGAGCCGTCTTTCCACC**	-51/-32
sg3	**CCAGGCCCCCGGCTCGGGGT**	-33/-14
sg4	**ACCTTCCCCATGGCTGGACA**	-9/+11

Supplementary Figure 1

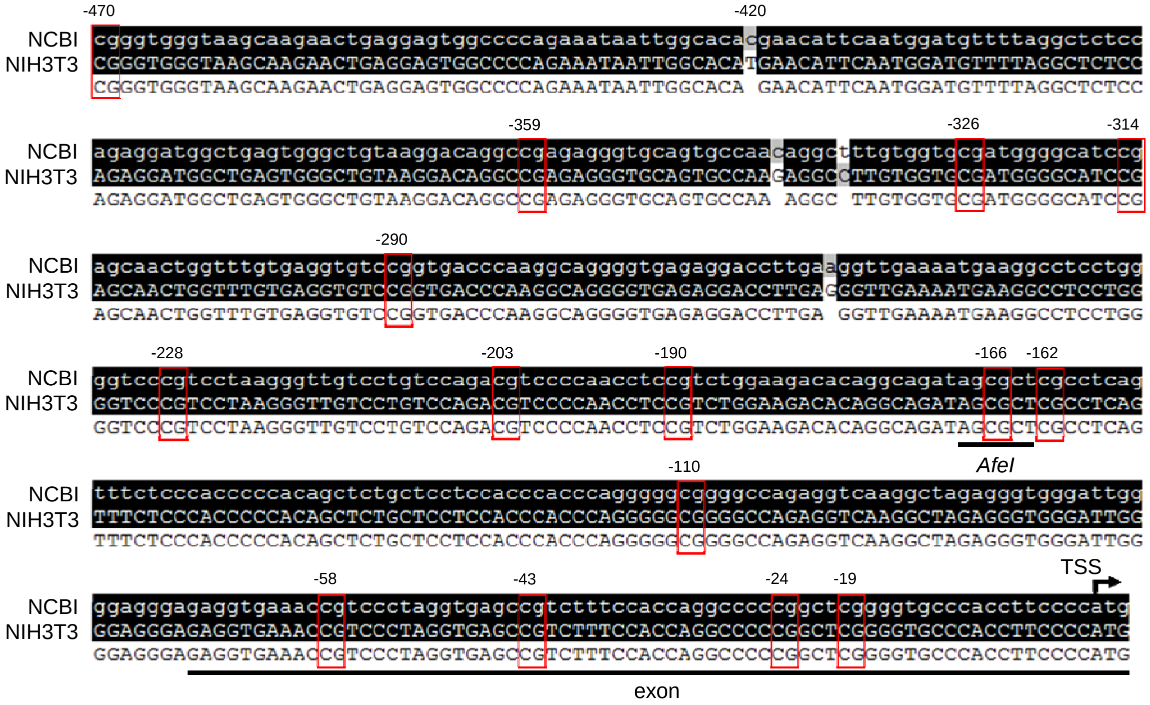

Supplementary Figure 2

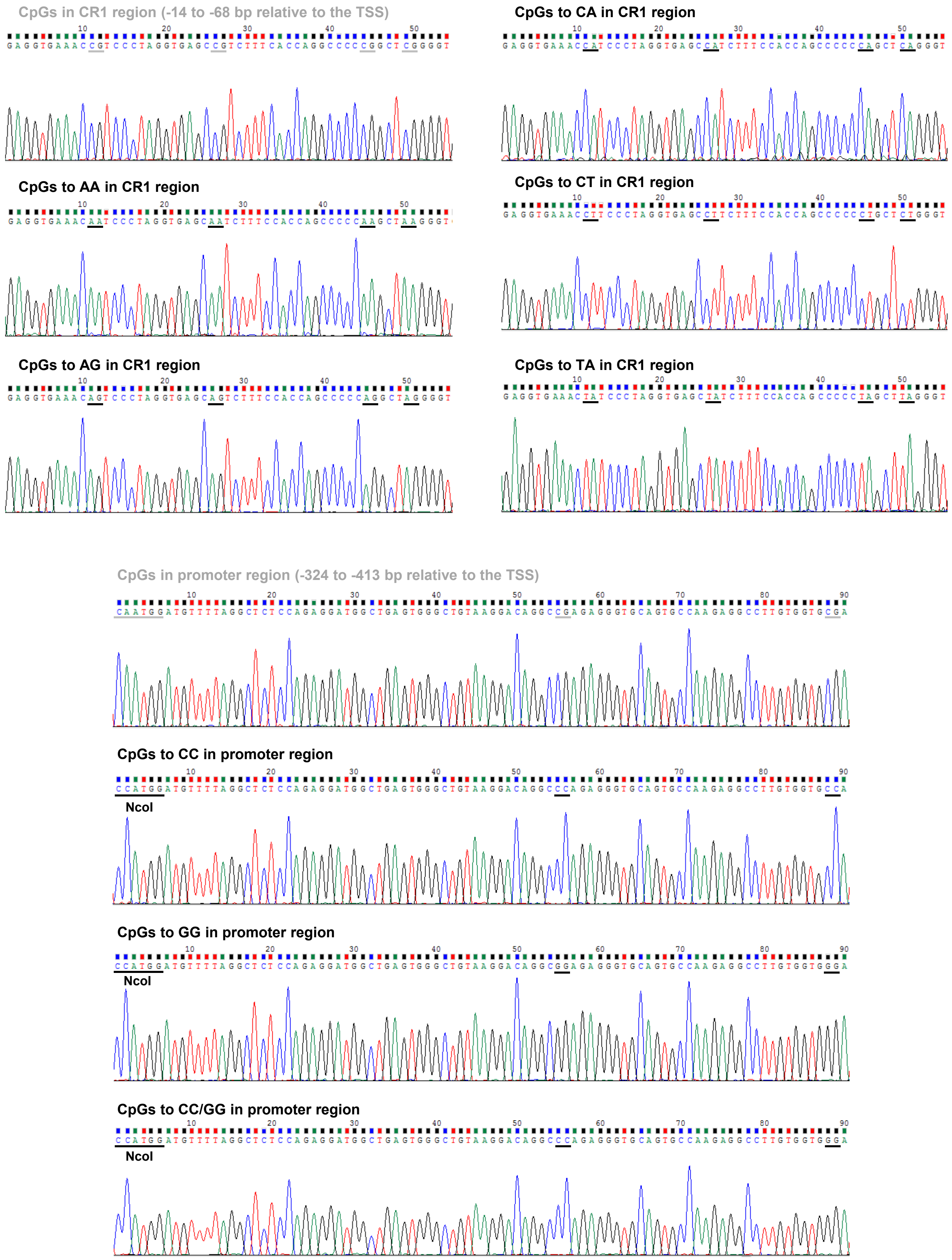

Supplementary Figure 3

a. -1 bp deletion

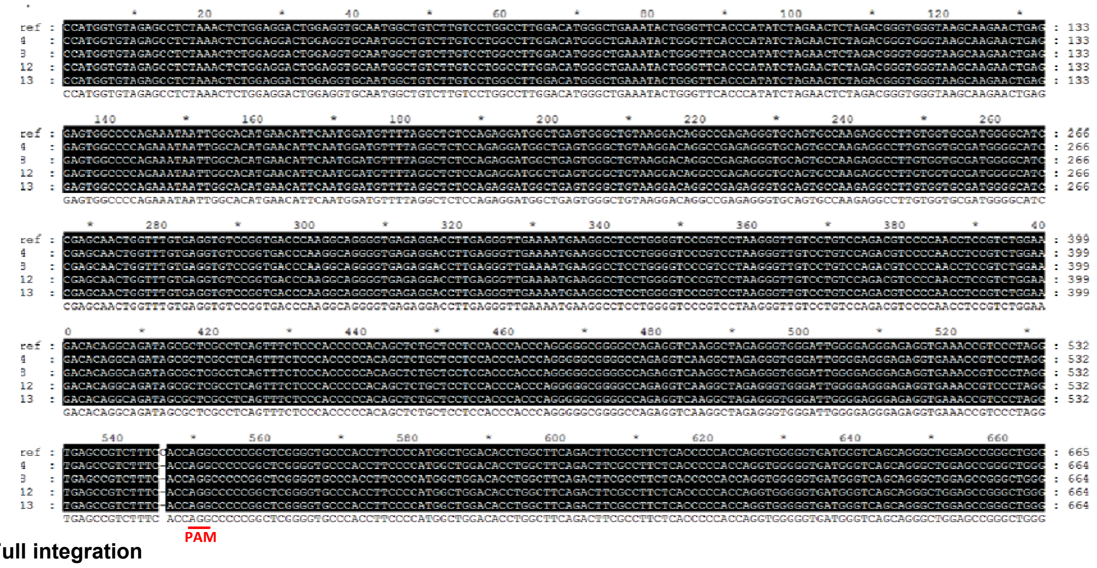

b. Full integration

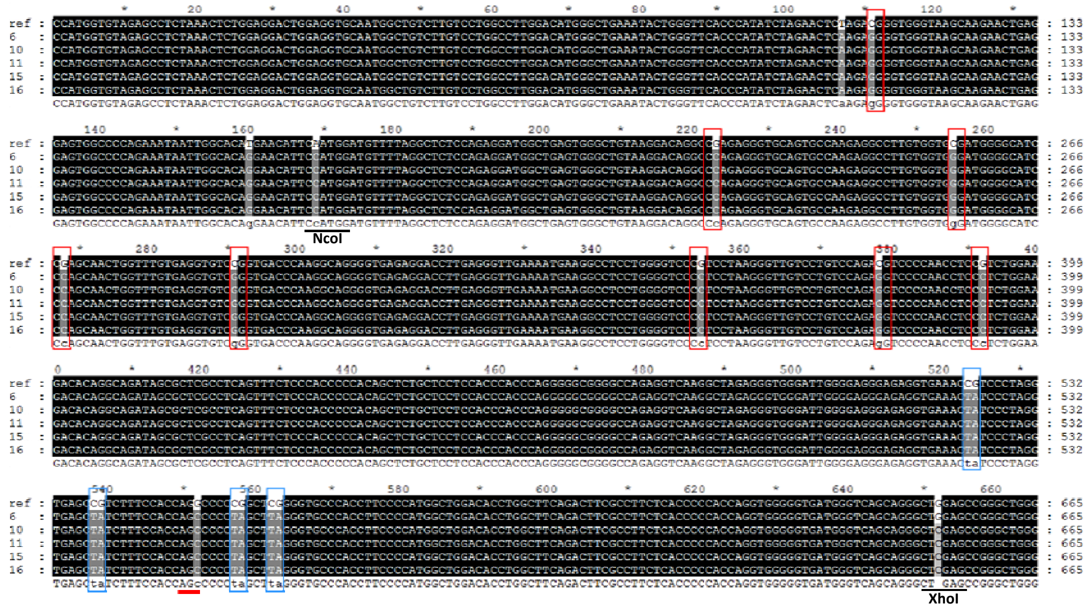

c. partial integration

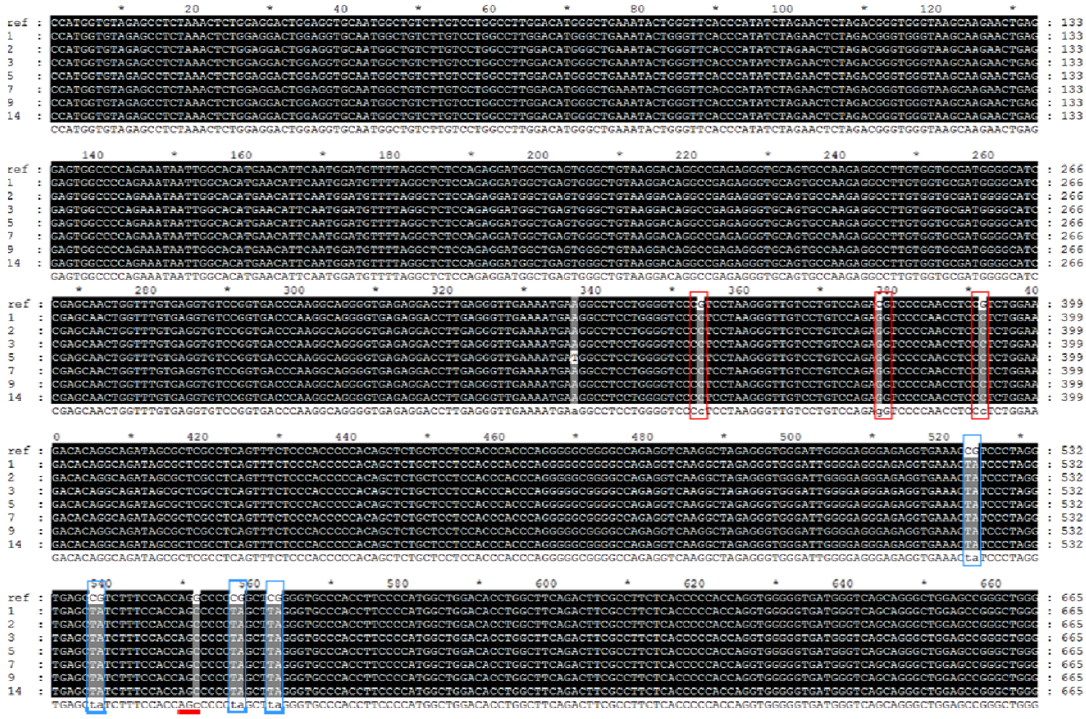

Supplementary Figure 4

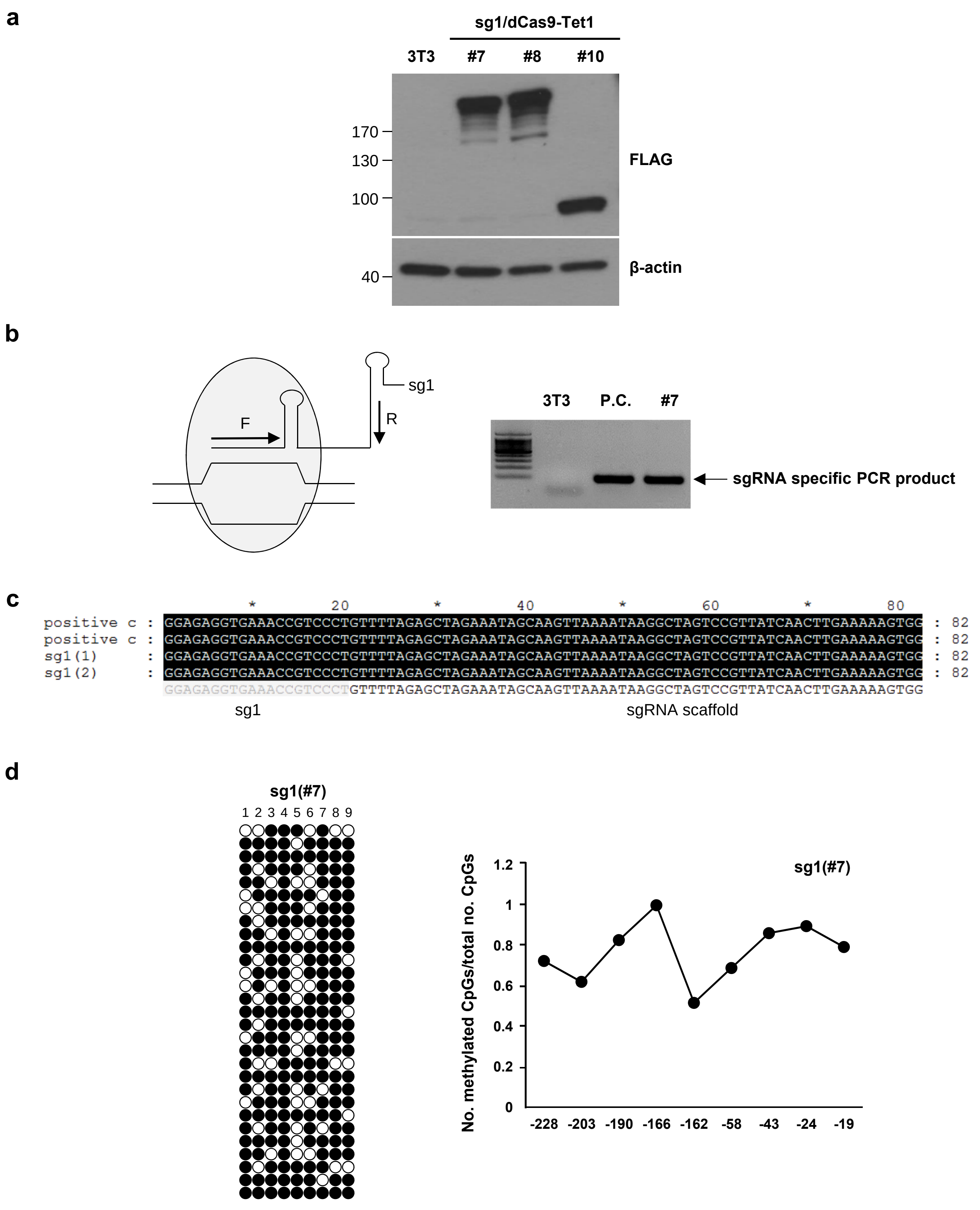

Supplementary Figure 5

Figure 5 (a)

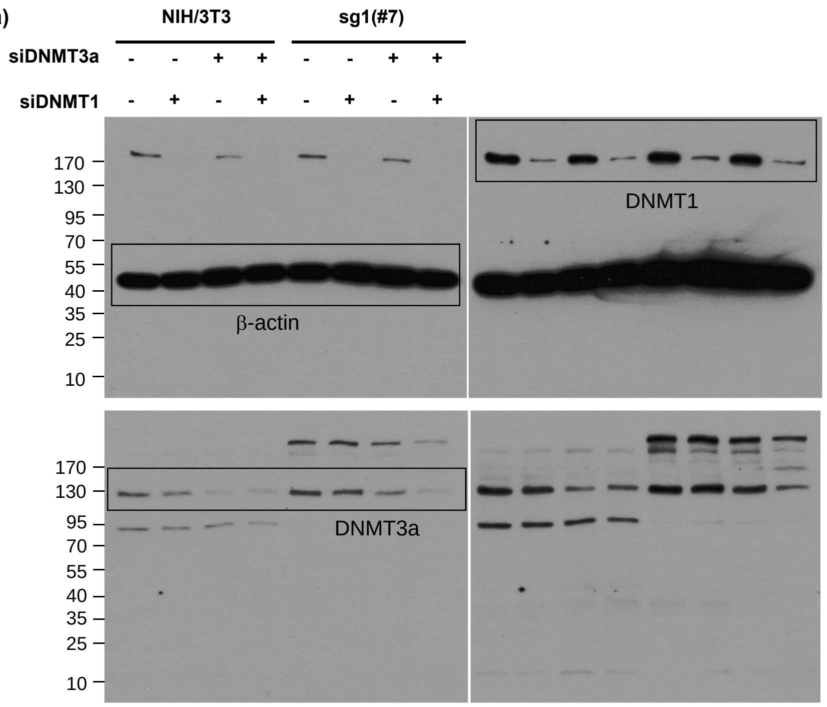

Figure 5 (d)

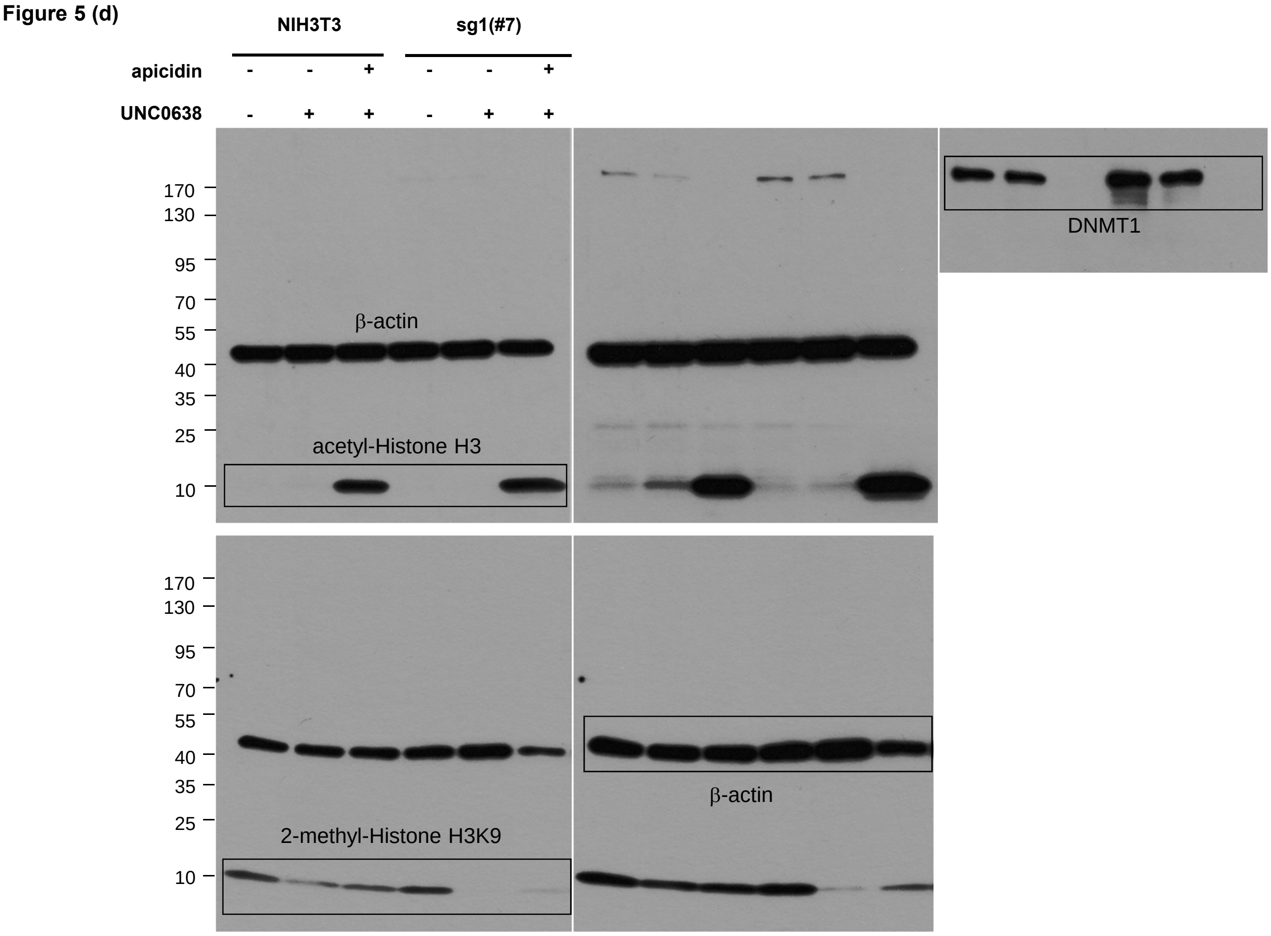

Supplementary Figure 6

Figure 5 (c)

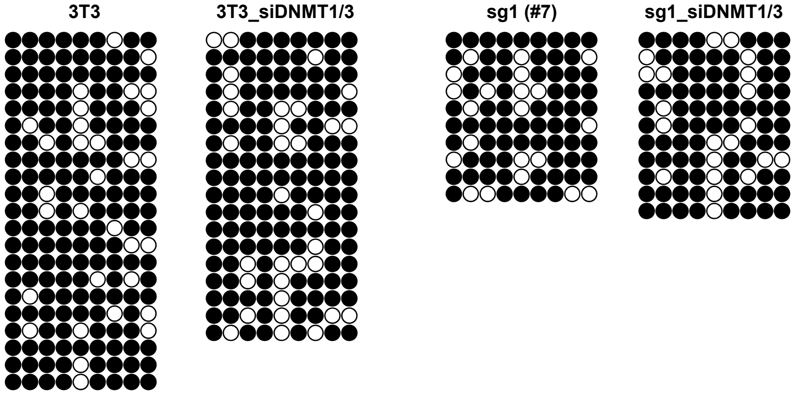

Figure 5 (f)

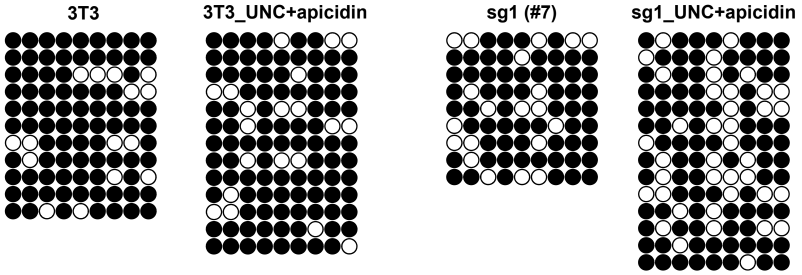

Supplement: Supplementary file 1 — Supplementary Information [file 41598_2019_48130_MOESM1_ESM.pdf]
